# Elevated risk of alcohol use disorder in Japanese men living with HIV: Psychosocial determinants and protective role of sense of coherence

**DOI:** 10.1016/j.dadr.2026.100437

**Published:** 2026-04-15

**Authors:** Taisuke Togari, Yoji Inoue, Gaku Oshima, Sakurako Abe, Rikuya Hosokawa, Yosuke Takaku

**Affiliations:** a Faculty of Liberal Arts, The Open University of Japan, Chiba, Japan; b Research and Development Department, Accelight Inc., Tokyo, Japan; c Department of Information and Communication, Meiji University, Tokyo, Japan; d Human Resources Department, TIS Inc., Tokyo, Japan; e Graduate School of Nursing for Health Care Science, Kyoto Prefectural University of Medicine, Kyoto, Japan; f Representative Director, Japanese Network of People living with HIV/AIDS, Tokyo, Japan

**Keywords:** People living with HIV, Alcohol use disorder, Sense of coherence, Social support

## Abstract

**Background:**

Men living with HIV face elevated risks for alcohol use disorder (AUD), yet few studies have examined sociodemographic and psychosocial correlates of AUD risk in Asian populations. Sense of coherence (SOC), reflecting the ability to cope with stress, may serve as a protective factor against AUD. This study investigated factors associated with AUD risk among men living with HIV in Japan.

**Methods:**

This cross-sectional study analyzed data from the third HIV Futures Japan nationwide survey (November 2019–July 2020). Of 908 respondents, 883 biologically male participants who completed alcohol use disorder screening were analyzed. AUD status was classified as low-risk drinking, at-risk drinker, or suspected AUD. Multinomial logistic regression examined sociodemographic and psychosocial predictors.

**Results:**

Among participants, 46.8% were classified as low-risk drinking, 44.7% as at-risk drinkers, and 8.5% as suspected AUD. Compared with the low-risk drinking group, heterosexual orientation (OR: 0.38, 95% CI: 0.17–0.86), higher education (OR: 0.63, 95% CI: 0.43–0.90), and older age (OR: 0.98, 95% CI: 0.96–1.00) were associated with lower odds of at-risk drinking. No sociodemographic factors associated with suspected AUD. In the fully adjusted psychosocial model, higher SOC was associated with lower odds of both at-risk drinking (OR: 0.98, 95% CI: 0.96–0.99) and suspected AUD (OR: 0.96, 95% CI: 0.93–0.98).

**Conclusions:**

The prevalence of suspected AUD among men living with HIV in Japan (8.5%) was substantially higher than in the general Japanese population (0.5%). SOC emerges as a robust protective factor, suggesting that interventions strengthening SOC may help mitigate AUD risk in this vulnerable population.

## Highlights

• 53.2% of Japanese men living with HIV showed at-risk or suspected AUD status.
• Lower sense of coherence consistently associated with elevated AUD risk.
• Mental health associations attenuated when sense of coherence was included.
• Gay men and those with lower education more likely to be at-risk drinkers.
• First study linking sense of coherence to AUD in Asian HIV populations.

## 1 Introduction

The global population of people living with HIV (PLWH) is estimated at 40.8 million (UNAIDS, 2025). The cumulative number of people with HIV and AIDS in Japan from 1985 to 2024 was 25,194 (22,523 men and 2671 women) and 11,181 (10,252 men and 929 women), respectively (Disease, 2025). Notably, men account for approximately 89% of PLWH in Japan, with men who have sex with men (MSM) representing the predominant transmission route. As HIV is a chronic condition without a cure, this substantial population of PLWH continues to require comprehensive long-term care and support to maintain health and quality of life.

Substance use disorder substantially affects HIV care and treatment outcomes in PLWH (Hall et al., 2013). Among PLWH in the United States, the prevalence of substance use disorder is reportedly 48% with alcohol use disorder (AUD) being the second most common at 19% following marijuana (Hartzler et al., 2017). Systematic review and meta-analyses have reported estimated AUD prevalence rates of 42.1% in developed countries, 24.5% in developing countries (Duko et al., 2019a), and 22.0% in African countries (Necho et al., 2020). In Ethiopia, a systematic review found prevalence rates of 36.42%, 19.00%, and 21.64% for lifetime, current, and hazardous alcohol use among PLWH, respectively (Mekuriaw et al., 2021). For comparison, the prevalence of AUD in the general population has been reported as 10.5% in the United States (National Institute on Alcohol Abuse and Alcoholism, 2023), indicating that PLWH face substantially elevated AUD risk. In Japan, where the HIV epidemic predominantly affects men, focused research on AUD among men living with HIV is particularly important. AUD poses significant clinical challenges for PLWH: it impairs adherence to antiretroviral treatment (Azar et al., 2010, Bach Xuan et al., 2014, Bizuneh et al., 2024, Chander et al., 2006, Neuman et al., 2012) and is associated with lower CD4 cell counts and higher viral loads (Baum et al., 2010, Chander et al., 2006). Although psychotherapy is sometimes provided to PLWH with AUD, treatment outcomes are often suboptimal (Madhombiro et al., 2019).

Research in the general population has identified multiple factors associated with AUD development, many of which may be relevant to understanding AUD among PLWH. Social determinant factors include low socioeconomic status (Schou and Moan, 2016) and young age. In addition, recent studies have found that being a sexual minority affects AUD development (Kahle et al., 2020, Shechory Bitton and Noach, 2022). Poor social support (Chen, 2006), narrow social networks (Kahle et al., 2020, Mikkelsen et al., 2015), and low social capital (Larm et al., 2016) are also associated with AUD. Many studies investigating the relationship between AUD and stress have demonstrated that AUD is strongly associated with post-traumatic stress disorder (Smith and Cottler, 2018), and there is evidence that depression and anxiety cause alcohol dependence (Alegría et al., 2010, Rudenstine et al., 2020). Moreover, high resilience to stress reportedly suppresses the development of AUD(Long et al., 2017) and AUD recurrence (Yamashita and Yoshioka, 2016).

Studies on the social determinants of AUD in PLWH have been conducted primarily in African countries, reporting associations with low income status (Bultum et al., 2018, Duko et al., 2019a, Necho et al., 2020), low education level (Bultum et al., 2018, Duko et al., 2019a), and poor social support(Duko et al., 2019b; Necho et al., 2020) in Africa. However, findings regarding educational level have been inconsistent, with at least one study reporting a weak inverse association(Ghaimo et al., 2025). An international systematic review of studies conducted in the United States, Canada, and China found no association between perceived stigma and AUD (Armoon et al., 2022). Although previous studies have reported associations between AUD and psychosocial factors such as social support, depression, anxiety, and stress resilience in the general population, research on these factors among PLWH in developed countries remains scarce.

In Japan, the current status of substance use disorder and AUD in PLWH remains unclear. A 2014 nationwide survey reported that 12.8% of PLWH were suspected of having AUD (The_Futures_Japan_Project, 2015), substantially higher than the 0.5% prevalence in the general Japanese population (Ministry of Health, 2021). Despite this elevated prevalence, no studies have investigated the psychosocial and socioeconomic determinants of AUD among PLWH in Japan.

Among these psychosocial factors, sense of coherence (SOC) has gained increasing attention as a potential protective factor against AUD. The SOC, a core concept of Antonovsky‘s salutogenic model, is a way of perceiving and engaging with one's environment and life, consisting of three constructs: sense of comprehensibility, sense of manageability, and sense of meaningfulness (Antonovsky, 1979). SOC is considered a key determinant of health, well-being and quality of life (Eriksson and Lindström, 2006; Eriksson and Lindström, 2007). A meta-analysis of eight studies examining SOC and alcohol use in the general population found that high SOC was associated with lower odds of alcohol use (pooled OR = 0.70), although with low certainty of evidence (Danigno et al., 2024). However, individual study findings have been inconsistent; one study found that SOC strongly suppressed hazardous alcohol use (Larm et al., 2016), another study demonstrated that it was not associated with the severity of alcohol addiction (Arévalo et al., 2008). Despite the potential relevance of SOC to AUD prevention, no studies have examined the relationship between SOC and AUD among PLWH worldwide, particularly in Japan where both AUD prevalence is elevated and psychosocial determinants remain unexplored.

This study aimed to examine the prevalence of AUD and at-risk drinking among Japanese men living with HIV, and to identify psychosocial and socioeconomic determinants associated with elevated AUD risk, with a particular focus on the protective role of SOC. Given the absence of research on psychosocial determinants of AUD among PLWH in Japan, these findings will provide essential evidence for developing targeted prevention and intervention strategies for this population.

## 2 Methods

### 2.1 Study design and participants

This cross-sectional, observational study used data from the third HIV Futures Japan nationwide online survey of PLWH residing in Japan. The Futures Japan nationwide surveys were planned and conducted by 20 representatives of support groups for PLWH in Japan and 20 researchers. A questionnaire was developed for the surveys, which used a participatory research methodology (Jagosh et al., 2012). Fifteen non-profit organizations supporting PLWH across Japan were involved in the planning and operation of the Futures Japan project (which involved the prefectures of Hokkaido, Aomori, Sendai, Niigata, Tokyo, Nagoya, Kyoto, Osaka, Hiroshima, Fukuoka, and Okinawa) and their core members recruited participants mainly by snowball sampling using websites and social networking services. As there is no national HIV registry system in Japan, most surveys of PLWH are conducted through hospital outpatient clinics, which limits participation to those regularly attending medical facilities. In contrast, the HIV Futures Japan surveys use community-based recruitment through support organizations, allowing participation from PLWH regardless of their current healthcare engagement status. This approach has the advantage of potentially including individuals who are HIV-positive but are not currently attending hospital.

**Fig. 1:**
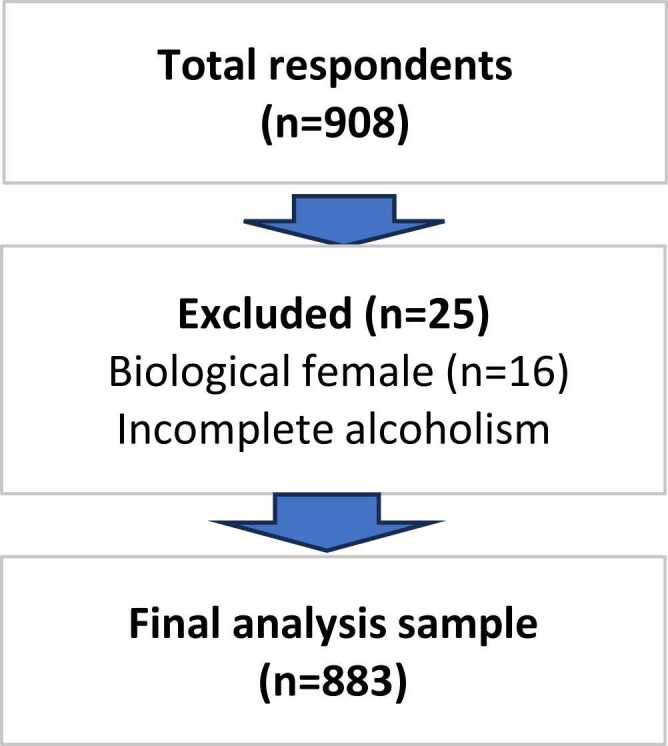
Flow diagram of participant selection.

The third survey was conducted from November 2019 to July 2020, and responses from 908 respondents were considered valid. Of these, 25 respondents were excluded: 16 were biological females and 9 had incomplete alcohol use disorder screening data. The final analysis included 883 biologically male participants who completed the alcohol use disorder screening test (Fig. 1).

Logistic regression analysis was used in this study. The sample size for logistic regression analysis is based on the number of independent variables (Peduzzi et al., 1996). The calculated required sample size for this study was 220 participants or more; thus, our sample size was considered adequate.

This study was approved by the Institutional Review Board of the Open University of Japan (approval number: 2019–35, approved on November 7, 2019) and conducted in accordance with the Declaration of Helsinki and the Ethical Guidelines for Medical Research Involving Human Subjects (Ministry of Education, Culture, Sports, Science and Technology and Ministry of Health, Labour and Welfare, Japan). Prior to participation, all potential participants were provided with information regarding the study purpose, procedures, voluntary participation, right to withdrawal, and data confidentiality. Informed consent was obtained electronically via a mandatory confirmation button. Written signatures were not required in accordance with Japanese ethical guidelines for observational research. Survey responses were collected without directly identifiable personal information, and data were securely stored with access limited to research team members.

### 2.2 Variables

#### 2.2.1 Risk of AUD

The revised version of the Kurihama Alcoholism Screening Test (KAST) was used. The KAST was developed in 1978, and is frequently used in screenings for AUD and epidemiological surveys in Japan (Saito and Ikegami, 1978). The KAST was further revised in 2004 to increase its accuracy. Surveys in Japan have demonstrated that the KAST is more sensitive in discriminating AUD than the Alcohol Use Disorders Identification Test and the CAGE Alcohol Questionnaire for the Japanese population (Osaki et al., 2016). The KAST contains 10 yes/no questions for men. We considered scores of ≥ 4 as indicating suspicion of AUD (s/o AUD), scores of 1–3 as indicating at-risk drinkers (potential AUD risk), and a score of 0 as low-risk drinking (Higuchi, 2005).

#### 2.2.2 Depression and anxiety tendency

The Japanese version of the Hospital Anxiety and Depression Scale (HADS) was used (Hatta et al., 1998). The HADS was developed by Zigmond and Snaith for patients with somatic symptoms, and the reliability and validity of its Japanese version have been confirmed (Zigmond and Snaith, 1983). The HADS consists of 7 items on depressive tendency (HADS-D) and 7 items on anxious tendency (HADS-A). Each item was evaluated on a 4-point scale (0–3) and the total score was used for analysis. In the present study, Cronbach’s alpha was 0.86 for the anxiety subscale and 0.76 for the depression subscale.

#### 2.2.3 Social support

The modified version of the Medical Outcomes Study Social Support Survey (mMOS-SS) is a self-administered questionnaire about social support developed for patients. Moser et al. shortened the 19-item version, which was designed to assess the functional and social support of local patients with chronic disease, to an 8-item version consisting of 4 items on instrumental support and 4 items on emotional support, and confirmed the reliability and validity of the 8-item version (Moser et al., 2012). The reliability and validity of the Japanese version of the mMOS-SS have also been confirmed (Togari and Yokoyama, 2016). Each item is evaluated on a 5-point scale: “none of the time,” “a little of the time,” “some of the time,” “most of the time,” and “all of the time.” In the present study, Cronbach’s alpha was 0.95 for the instrumental support subscale and 0.92 for emotional support subscale.

#### 2.2.4 Sense of coherence

The 13-item version of the SOC scale (SOC-13) was used. The SOC-13 measures SOC, developed by Antonovsky (Antonovsky, 1993). The reliability and validity of the Japanese version of the SOC-13 have been confirmed (Togari et al., 2008). The SOC-13 contains 13 items on a 7-point scale. Cronbach’s alpha for the scale was 0.73 in this study. The total score ranges from 13 to 91, with higher scores indicating stronger SOC. SOC was analyzed as a continuous variable in this study.

#### 2.2.5 Socioeconomic status

The evaluation of socioeconomic status was based on participants’ educational background and annual personal income. Educational background was classified into three categories: high school graduate or lower (≤12 years), junior college/vocational school/college of technology (14–15 years), and college/graduate school (≥16 years). Annual personal income was classified into six categories: less than $7,000 (less than 1.00 million yen), $7,000 to $21,000 (1.00–2.99 million yen), $21,000 to $36,000 (3.00–4.99 million yen), $36,000 to $57000 (5.00–7.99 million yen), $57000 or more (8.00 million yen or more), and unknown/no answer.

#### 2.2.6 Sexuality

Participants’ sexuality was categorized as heterosexual, gay, bisexual, and other/unknown.

#### 2.2.7 Regular hospital attendance

The participants were asked whether they had “regularly attended hospital for the treatment of HIV/AIDS in the last year,” and their responses were classified into two categories: “attended hospital” or “did not attend hospital.”

#### 2.2.8 Attributes

Participants were asked about their age, number of family members living together (“≥2” or “living alone”), and number of years since the diagnosis of HIV. Age and the number of years since HIV diagnosis were considered continuous variables.

### 2.3 Analytic methods

To investigate the association of the risk of AUD with sociological attributes and socioeconomic status, multinomial logistic regression analysis was performed with three categories of AUD risk as the dependent variable and sexuality, age, the number of family members living together, regular hospital attendance, and the number of years since HIV diagnosis as independent variables. The reference category for the dependent variable was “low-risk drinking.” First, a bivariate analysis was performed with the dependent variable and one of the independent variables, then a multivariate analysis incorporating all independent variables was performed.

Hierarchical multinomial logistic regression analysis was performed to examine cumulative associations. Model 1included sociodemographic confounders plus social support variables (instrumental and emotional support), representing observable environmental resources. Model 2 added mental health variables (HADS-A and HADS-D scores), which may be influenced by both social support and individual coping capacity. Model 3 added SOC-13 score, conceptualized as a relatively stable cognitive orientation reflecting the capacity to integrate social support and mental health resources. This approach examined incremental contributions of each variable set, and whether SOC maintains its association after accounting for other psychosocial factors. Model fit was assessed using AIC and Nagelkerke R².

For both analytical approaches, missing data in psychosocial variables (instrumental support, emotional support, HADS-A score, HADS-D score, and SOC-13 score) were examined using Little’s chi-square test to investigate whether data were missing completely at random. The results were as follows: chi-square= 238.857, degrees of freedom= 254, and p = 0.744. As the missing data were missing completely at random, they were imputed using the expectation–maximization method for the analysis.

IBM SPSS statistics 26.0 (Armonk, NY, USA) was used for the analysis.

## 3 Results

The risk of AUD was low-risk drinking, at-risk drinker, and s/o AUD in 413 (46.8%), 395 (44.7%), and 75 (8.5%) participants, respectively. Table 1 shows the distribution of the participants in this study. In the overall sample, the most common sexuality was gay (85.5%), and 97.4% of the participants attended hospital regularly. The mean age (standard deviation) was 42.2 (9.2) years, and the mean (standard deviation) number of years since HIV diagnosis was 8.0 (6.6). The proportion of the participants who graduated from college/graduate school was 54.1%. Statistically significant differences across AUD risk categories were observed for anxious tendency (HADS-A; p = 0.005) and sense of coherence (SOC-13; p < 0.001), with higher anxiety and lower SOC associated with greater AUD risk.

**Table 1:** Sample characteristics by alcohol use disorder risk categories.

					The Kurihama alcoholism screening test											
		Overall (N = 883)			Low-risk drinking (n = 413)			At-risk drinkers (n = 395)			s/o AUD (n = 75)					
		n	(%)		n	(%)		n	(%)		n	(%)		p		
Sexuality														.050	^†^	
	Gay	755	(85.5)		349	(84.5)		337	(85.3)		69	(92.0)				
	Heterosexual	34	(3.9)		24	(5.8)		9	(2.3)		1	(1.3)				
	Bisexual	79	(8.9)		35	(8.5)		41	(10.4)		3	(4.0)				
	Others/unknown	15	(1.7)		5	(1.2)		8	(2.0)		2	(2.7)				
Hospital attendance														.451	^†^	
	Not attending hospital regularly	23	(2.6)		8	(1.9)		12	(3.0)		3	(4.0)				
	Attending hospital regularly	860	(97.4)		405	(98.1)		383	(97.0)		72	(96.0)				
The number of family members living together														.600	^†^	
	2 or more	434	(49.2)		202	(48.9)		191	(48.4)		41	(54.7)				
	1	449	(50.8)		211	(51.1)		204	(51.6)		34	(45.3)				
Educational background														.050	^†^	
	High school or lower	197	(22.3)		78	(18.9)		106	(26.8)		13	(17.3)				
	Professional training college/junior college/national colleges of technology	203	(23.0)		105	(25.4)		82	(20.8)		16	(21.3)				
	College/graduate school	478	(54.1)		229	(55.4)		203	(51.4)		46	(61.3)				
	Others	5	(0.6)		1	(0.2)		4	(1.0)		0	(0.0)				
Annual income														.653	^†^	
	Less than 1.00 million	51	(5.8)		21	(5.1)		25	(6.3)		5	(6.7)				
	1.00–2.99 million	256	(29.0)		126	(30.5)		111	(28.1)		19	(25.3)				
	3.00–4.99 million	282	(31.9)		128	(31.0)		132	(33.4)		22	(29.3)				
	5.00–7.99 million	186	(21.1)		88	(21.3)		84	(21.3)		14	(18.7)				
	8.00 million or more	81	(9.2)		40	(9.7)		30	(7.6)		11	(14.7)				
	Unknown/no answer	27	(3.1)		10	(2.4)		13	(3.3)		4	(5.3)				
Age, mean (SD)		42.2	(9.2)		42.8	(9.4)		41.7	(8.9)		41.0	(9.0)		.134	^‡^	
The number of years after being found to be HIV-positive, mean (SD)		8.0	(6.6)		8.0	(6.6)		8.1	(6.7)		7.4	(6.0)		.686	^‡^	
mMOS-SS																
	Instrumental support, mean (SD)	42.4	(35.2)		43.6	(34.8)		41.6	(35.7)		40.4	(34.9)		.623	^‡^	
	Emotional support, mean (SD)	47.9	(30.8)		48.4	(31.2)		48.6	(30.3)		41.8	(31.7)		.192	^‡^	
HADS																
	Anxious tendency, mean (SD)	7.4	(4.6)		6.9	(4.5)		7.8	(4.6)		8.4	(4.7)		.005	^‡^	
	Depression, mean (SD)	7.6	(4.1)		7.4	(4.2)		7.7	(4.0)		8.1	(4.1)		.280	^‡^	
SOC-13		52.7	(13.8)		55.0	(14.1)		51.3	(13.0)		47.7	(14.0)		< .001	^‡^	

AUD risk categories classified as: low-risk drinking (0 points), At-risk drinking (1–3 points), Suspected AUD (≥4 points) based on the Kurihama Alcoholism Screening Test (KAST).

† Chi-square test

‡ F test

s/o AUD: suspicious of alcohol use disorder

mMOS-SS: modified medical outcome survey - social support survey

HADS: hospital anxiety and depression scale

SOC-13: 13-item version of the sense of coherence scale

Table 2 shows the results of the multinomial logistic regression analysis of the associations of attributes and socioeconomic status with AUD risk. Regarding sexuality, the multivariate analysis comparing the at-risk drinkers and low-risk drinking group showed an odds ratio (OR) [95% confidence interval (CI)] for heterosexuality of 0.38 [0.17–0.86] (p = 0.019). Regarding educational background, the OR [95% CI] was 0.57 [0.38–0.88] (p = 0.010) for professional training college/junior college/college of technology (14–15 years) and 0.63 [0.43–0.90] (p = 0.012) for college/graduate school (≥16 years). The OR for age was 0.98 [0.96–1.00] (p = 0.019) and demonstrated a lower association as age increased. AUD risk showed no associations with any variables in the s/o AUD group.

**Table 2:** Multinomial logistic regression analysis of sociodemographic and socioeconomic factors associated with AUD risk categories.

		Bivariate analysis															Multivariate analysis^†^													
		At-risk drinkers							s/o AUD									At-risk drinkers							s/o AUD					
				95% CI							95% CI									95% CI							95% CI			
		OR	[lower, upper]				P		OR	[lower, upper]				P				OR	[lower, upper]				P		OR	[lower, upper]				P
Sexuality																														
	Gay	1.00							1.00									1.00							1.00					
	Heterosexual	0.39	[	0.18	0.85	]	.018		0.21	[	0.03	1.58	]	.130				0.38	[	0.17	0.86	]	.019		0.20	[	0.03	1.59	]	.129
	Bisexual	1.21	[	0.75	1.95	]	.426		0.43	[	0.13	1.45	]	.175				1.29	[	0.79	2.10	]	.302		0.43	[	0.13	1.45	]	.174
	Others/unknown	1.66	[	0.54	5.12	]	.380		2.02	[	0.39	10.64	]	.405				1.80	[	0.57	5.73	]	.319		2.37	[	0.44	12.77	]	.317
Hospital attendance																														
	Attending hospital regularly	1.00							1.00									1.00												
	Not attending hospital regularly	1.59	[	0.64	3.92	]	.318		2.11	[	0.55	8.14	]	.279				1.38	[	0.54	3.52	]	.499		1.81	[	0.44	7.35	]	.409
The number of family members living together																														
	2 or more	1.00							1.00									1.00							1.00					
	Single	0.98	[	0.74	1.29	]	.874		1.26	[	0.77	2.06	]	.360				0.93	[	0.70	1.24	]	.623		1.23	[	0.74	2.04	]	.434
Educational background																														
	High school or lower	1.00							1.00									1.00							1.00					
	Junior college*	0.58	[	0.38	0.87	]	.008		0.91	[	0.42	2.01	]	.824				0.57	[	0.38	0.88	]	.010		0.97	[	0.43	2.18	]	.943
	College/graduate school	0.65	[	0.46	0.92	]	.016		1.21	[	0.62	2.35	]	.583				0.63	[	0.43	0.90	]	.012		1.11	[	0.55	2.24	]	.762
	Others	2.94	[	0.32	26.85	]	.339											2.67	[	0.28	25.12	]	.390							
Annual income																														
	Less than 1.00 million	1.59	[	0.75	3.36	]	.227		0.87	[	0.27	2.82	]	.811				0.78	[	0.41	1.49	]	.451		0.76	[	0.25	2.29	]	.620
	1.00–2.99 million	1.18	[	0.69	2.01	]	.558		0.55	[	0.24	1.25	]	.153				0.88	[	0.46	1.67	]	.694		0.78	[	0.26	2.33	]	.658
	3.00–4.99 million	1.38	[	0.81	2.34	]	.241		0.63	[	0.28	1.40	]	.253				0.87	[	0.45	1.71	]	.692		0.77	[	0.24	2.42	]	.653
	5.00–7.99 million	1.27	[	0.73	2.23	]	.399		0.58	[	0.24	1.39	]	.220				0.76	[	0.35	1.64	]	.479		1.38	[	0.41	4.70	]	.602
	8.00 million or more	1.00							1.00									1.00							1.00					
	Unknown/no answer	1.73	[	0.67	4.49	]	.257		1.46	[	0.38	5.54	]	.583				1.10	[	0.39	3.13	]	.854		1.53	[	0.33	7.24	]	.589
Age		0.99	[	0.97	1.00	]	.095		0.98	[	0.95	1.01	]	.128				0.98	[	0.96	1.00	]	.019		0.97	[	0.94	1.00	]	.081
The number of years after being found to be HIV-positive		1.00	[	0.98	1.02	]	.865		0.99	[	0.95	1.02	]	.437				1.01	[	0.99	1.04	]	.288		1.00	[	0.96	1.05	]	.982

Dependent variable: Alcohol use disorder (AUD) risk categories. Reference category: Low-risk drinking group.

*junior college/vocational school/colleges of technology

†-2log likelihood: 1570.98, Nagelkerke R^2^:.055

OR: Odds ratio

s/o AUD: suspicious of alcohol use disorder

Table 3 presents the results of the hierarchical multinomial logistic regression analysis. Model 1, which included sociodemographic confounders and social support variables, showed no significant associations between social support and AUD risk. Model 2, which added mental health variables, revealed borderline significant associations for anxious tendency with both at-risk drinking (OR [95% CI] = 1.04 [1.00–1.07], p = 0.067) and s/o AUD (OR [95% CI] = 1.06 [1.00–1.13], p = 0.057). Model 3, which added SOC, demonstrated that higher SOC was associated with lower odds of both at-risk drinking (OR [95% CI] = 0.98 [0.96–0.99], p = 0.001) and s/o AUD (OR [95% CI] = 0.96 [0.93–0.98], p = 0.001). Model 3, the associations of anxiety and depression with AUD risk were substantially attenuated compared to Model 2 (anxiety: at-risk drinking OR=1.00 [0.96–1.04], p = 0.893; s/o AUD OR=1.00 [0.92–1.07], p = 0.905; depression: at-risk drinking OR=0.98 [0.94–1.03], p = 0.448; s/o AUD OR=0.97 [0.90–1.05], p = 0.461). Model 3 showed the best fit among the three models (AIC = 1576.7, Nagelkerke R² = 0.095).

**Table 3:** Hierarchical multinomial logistic regression analysis of psychosocial variables associated with AUD risk.

	Model 1						Model 2						Model 3					
	At-risk drinkers			s/o AUD			At-risk drinkers			s/o AUD			At-risk drinkers			s/o AUD		
	95% CI			95% CI			95% CI			95% CI			95% CI			95% CI		
Independent variable	OR	[lower, upper]	P	OR	[lower, upper]	P	OR	[lower, upper]	P	OR	[lower, upper]	P	OR	[lower, upper]	P	OR	[lower, upper]	P
Instrumental support	1.00	[0.99, 1.00]	.494	1.00	[0.99, 1.04]	.821	1.00	[0.99, 1.00]	.466	1.00	[0.99, 1.01]	.691	1.00	[0.99, 1.00]	.523	1.00	[0.99, 1.01]	.641
Emotional support	1.00	[1.00, 1.01]	.359	0.99	[0.98, 1.00]	.056	1.00	[1.00, 1.01]	.513	0.99	[0.98, 1.00]	.087	1.00	[1.00, 1.01]	.333	0.99	[0.98, 1.00]	.154
Anxious tendency (HADS-A)							1.04	[1.00, 1.07]	.067	1.06	[1.00, 1.13]	.057	1.00	[0.96, 1.04]	.893	1.00	[0.92, 1.07]	.905
Depressive tendency (HADS-D)							1.00	[0.96, 1.05]	.923	1.00	[0.93, 1.08]	.907	0.98	[0.94, 1.03]	.448	0.97	[0.90, 1.05]	.461
Sense of coherence (SOC-13)													0.98	[0.96,.99]	.001	0.96	[0.93, 0.98]	.001
Nagelkerke R²			.064						.074						.095			
AIC			1637.66						1691.491						1618.736			

Dependent variable: Alcohol use disorder (AUD) risk categories. Reference category: Low-risk drinking group.

Model 1: Sociodemographic variables + social support (instrumental and emotional)

Model 2: Model 1 + mental health (anxiety and depression)

Model3: Model 2 + sense of coherence

s/o AUD, suspicious of alcohol use disorder: soc13: 13-item version of the sense of coherence scale; AIC, Akaike information criterion

## 4 Discussion

This study demonstrated the prevalence of suspected AUD among Japanese men living with HIV was 8.5%, substantially higher than that in the general Japanese population (0.5%) (Ministry of Health, 2021), corresponding to a prevalence ratio of 17.0. It should be noted that while our study used the KAST screening tool, the general population estimate may have been based on different assessment methods, which could affect direct comparability. This prevalence observed in our study was lower than those reported for people living with HIV in the United States (19%) (Hartzler et al., 2017) and Africa (22%) (Necho et al., 2020), although caution is warranted when comparing across studies that may employ different diagnostic instruments and criteria. Notably, when comparing prevalence ratios with the general population, the disparity in Japan (17.0) was more pronounced than that in the United States (1.9, with a general population prevalence of 10.5% (National Institute on Alcohol Abuse and Alcoholism, 2023)). This marked disparity between men living with HIV and the general population in Japan underscore AUD as a significant public health concern among PLWH in Japan and highlights the importance of understanding its psychosocial and socioeconomic determinants.

Regarding sexual orientation, AUD risk tended to be low in heterosexual participants and high in gay participants. A similar trend has been observed in the general population(Kahle et al., 2020; Shechory Bitton and Noach, 2022), suggesting that the elevated AUD risk among sexual minority men may not be specific to those living with HIV. This finding confirms elevated AUD risk among gay men living with HIV compared with their heterosexual counterparts. In Japan, behavioral science research on sexual minority populations remains limited, and further research is needed to elucidate the mechanisms underlying this disparity in the Japanese context.

Regarding socioeconomic factors, participants with fewer years of education showed higher AUD risk, consistent with findings from previous studies of PLWH in Africa (Bultum et al., 2018, Duko et al., 2019a). In contrast, annual income showed no association with AUD risk in this study, differing from findings in African settings, particularly Ethiopia, where lower income was associated with elevated AUD risk (Bultum et al., 2018, Duko et al., 2019a, Necho et al., 2020). The persistent association with education but not income suggests that educational attainment may capture aspects of health literacy or coping skills that influence alcohol use behaviors independently of economic resources. The absence of an income-AUD association in Japan may reflect differences in drinking culture between Japan and the settings where previous studies were conducted, particularly regarding alcohol use patterns among higher-income individuals.

Regarding social support, higher emotional support was associated with suspected AUD, instrumental support showed no clear association with AUD risk. This finding contrasts with a study by Duko et al. which reported that social support was association with lower AUD risk in PLWH (Duko et al., 2019b). The inconsistency may reflect differences in the dimensions of social support assessed, population characteristics, or cultural context. Instrumental support showed no clear association with AUD risk. It should be noted that the present study assessed only emotional and instrumental support using a brief scale; other dimensions of social support, such as informational, appraisal, or belongingness support, were not examined and may have different relationships with AUD risk.

Regarding mental health, higher anxiety was associated with higher AUD risk, while the association with depression was less consistent. The more robust association with anxiety compared to depression suggests that anxious arousal or worry may be more directly linked to alcohol use behaviors among men living with HIV in Japan than depressive symptoms. These findings are generally consistent with evidence from the general population linking psychological distress to AUD development (Alegría et al., 2010, Rudenstine et al., 2020). However, in the hierarchical models, the associations of both anxiety and depression with AUD risk were substantially attenuated when sense of coherence was included, suggesting that these relationships may be partly explained by broader coping orientations.

Regarding sense of coherence, higher SOC was consistently associated with lower AUD risk. Notably, in the hierarchical models, when SOC was added, the associations of anxiety, depression, and emotional support with AUD risk were substantially attenuated, while SOC maintained its association with AUD risk. This pattern suggests that SOC may play a more fundamental role in AUD risk than these other psychosocial factors, potentially representing a common underlying dimension of psychological resilience. Previous studies in the general population have reported inconsistent findings, with some demonstrating an association between SOC and AUD (Larm et al., 2016) and others showing no association (Arévalo et al., 2008). The present study extends evidence for this association to Japanese men living with HIV. The development of AUD is substantially affected by stress early in life (Moustafa et al., 2021). Although SOC develops after birth, it is considered a relatively stable ability for coping with stress (Antonovsky, 1987), and individuals with higher SOC are generally less susceptible to stress (Eriksson and Lindström, 2006). Therefore, SOC may serve as a protective factor against the development of AUD among PLWH. The robustness of the SOC-AUD association across different model specifications, and its apparent role in explaining associations of other psychosocial variables, suggests that strengthening SOC should be considered a priority in mental health interventions for this population.

The present study had several limitations. First, the survey used snowball sampling to obtain participants, mainly through PLWH support groups in Japan. Because Japan does not have a national HIV registry system, the reproducibility of these findings should be evaluated using samples from diverse sources, including HIV prevention education groups and public health centers. Second, we did not account for gender as a potential confounding factor. Although biological sex was recorded, no information was collected about transgender status, which may substantially affect AUD development. Third, all measures were based on self-report, which may be subject to social desirability bias and could lead to underreporting of alcohol consumption. Additionally, AUD status was determined using a screening instrument (KAST) rather than clinical diagnostic interviews, which may have affected the accuracy of case identification.

Several methodological considerations regarding the psychosocial analyses warrant discussion. The hierarchical modeling approach revealed substantial attenuation of associations for anxiety, depression, and emotional support when SOC was included. This pattern could reflect mediation, shared variance among theoretically interrelated constructs, or overadjustment bias. The cross-sectional design prevents definitive determination of causal relationships and temporal ordering among these psychosocial factors. Future longitudinal research is needed to clarify the causal pathways between psychosocial factors and AUD development

This study demonstrated that AUD prevalence among Japanese men living with HIV is substantially elevated compared with the general population. Among psychosocial factors, SOC showed the most robust association with AUD risk, maintaining its association when other psychosocial variables were accounted for. Lower educational attainment and gay sexual orientation were also associated with higher AUD risk. These findings suggest that strengthening SOC may be important for AUD prevention among PLWH, and highlight the need for targeted prevention approaches among vulnerable subgroups, particularly gay men and those with lower educational attainment.

## CRediT authorship contribution statement

**Sakurako Abe:** Writing – review & editing, Methodology, Investigation, Data curation, Conceptualization. **Gaku Oshima:** Writing – review & editing, Validation, Data curation, Conceptualization. **Yoji Inoue:** Writing – review & editing, Validation, Supervision, Resources, Project administration, Methodology, Investigation, Data curation. **Yosuke Takaku:** Validation, Supervision, Conceptualization. **Rikuya Hosokawa:** Validation, Methodology, Investigation, Data curation, Conceptualization. **Taisuke Togari:** Writing – review & editing, Writing – original draft, Supervision, Software, Resources, Methodology, Investigation, Funding acquisition, Formal analysis, Data curation, Conceptualization.

## Declaration of Generative AI and AI-assisted technologies in the writing process

During the preparation of this work the authors used Claude Sonnet 4.5 in order to English proofreading. After using this tool/service, the authors reviewed and edited the content as needed and takes full responsibility for the content of the published article.

## Funding

This research was funded by Japan Society for the Promotion of Science KAKENHI, grant numbers 19H03928 and 25K21920.

## Declaration of Competing Interest

The authors declare that they have no known competing financial interests or personal relationships that could have appeared to influence the work reported in this paper.

## Data Availability

The data that support the findings of this study are available from the corresponding author upon reasonable request. The data are not publicly available due to privacy and ethical restrictions concerning sensitive information about people living with HIV.
